# The BfmRS stress response protects *Acinetobacter baumannii* against defects in outer membrane lipoprotein biogenesis

**DOI:** 10.1128/jb.00332-24

**Published:** 2024-12-11

**Authors:** Julianna Marotta, Alan Zhao, Philip N. Rather, Marcin Grabowicz

**Affiliations:** 1 Microbiology and Molecular Genetics Program, Graduate Division of Biological and Biomedical Sciences, Emory University, Laney Graduate School 310202 , Atlanta, Georgia, USA; 2 Department of Microbiology and Immunology, Emory University School of Medicine 12239 , Atlanta, Georgia, USA; 3 Division of Infectious Diseases, Department of Medicine, Emory University 234195 , Atlanta, Georgia, USA; 4 Emory Antibiotic Resistance Center, Emory University School of Medicine 12239 , Atlanta, Georgia, USA; 5 Research Service, Atlanta VA Medical Center 19998 , Decatur, Georgia, USA; University of Notre Dame, Notre Dame, Indiana, USA

**Keywords:** outer membrane, lipoproteins, *Acinetobacter baumannii*, cell envelope, stress responses, lipoprotein trafficking, BfmR, BaeR

## Abstract

The outer membrane (OM) of Gram-negative bacteria is the outermost layer of the cell and serves as permeability barrier against environmental toxins, including antibiotics. The OM is built by several pathways that transport and assemble lipids and proteins into the OM. Since the OM is an essential organelle for the cell, envelope stress responses (ESRs) continuously monitor its assembly to preserve viability if defects arise. While ESRs have been extensively characterized in *Escherichia coli*, they are generally narrowly conserved. Lipoprotein trafficking to the OM via the “Lol” pathway is a linchpin for all OM assembly pathways. In *E. coli*, defects in this essential process are sensed when the sensor OM lipoprotein NlpE activates the CpxAR two-component system. Distantly related *Acinetobacter baumannii* encodes an NlpE homolog but lacks any Cpx homolog; how OM lipoprotein stress might be sensed and mitigated in these bacteria is therefore unclear. Here, we used CRISPRi to transiently induce defects in OM lipoprotein synthesis (targeting *lgt* and *lnt*) or trafficking (targeting *lolA*) in *A. baumannii*. We defined the transcriptional response to blocks in OM lipoprotein biogenesis. After scrutinizing candidate ESRs, we identified the BfmRS two-component systems as specifically critical for preserving *A. baumannii* viability during stress in OM lipoprotein biogenesis. Surprisingly, *A. baumannii* NlpE played no role in combatting OM lipoprotein stress. Our study identifies an *A. baumannii* ESR for OM lipoprotein biogenesis defects that acts in a distinct mechanism, not involving the NlpE sensor lipoprotein.

**IMPORTANCE:**

As the cell’s surface, the outer membrane (OM) of bacteria, such as *Acinetobacter baumannii*, is continuously under assault from the environment or host. OM integrity is needed for cell survival, and envelope stress responses (ESRs) act to detect and repair any defects. ESRs are well-defined in *Escherichia coli* but are poorly conserved. We sought to identify an ESR for the essential process of OM lipoprotein biogenesis in *A. baumannii*. We found that the BfmRS two-component system performs this function and does so without relying on its NlpE sensor homolog, suggesting a novel mechanism of stress sensing is involved in *A. baumannii*. Our work identifies a key cellular role for BfmRS.

## INTRODUCTION

**Fig 1:**
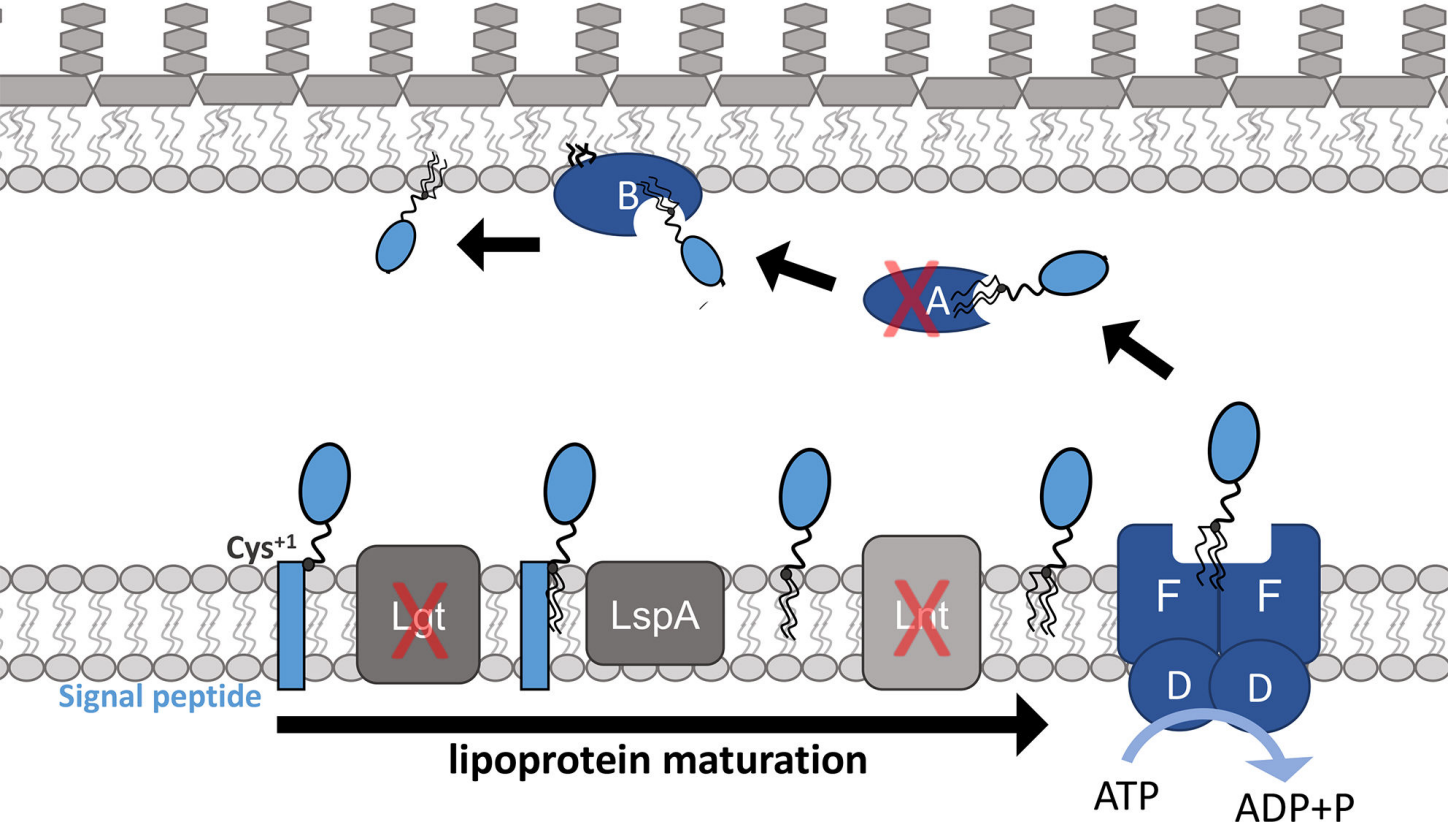
Targeting OM lipoprotein biogenesis in *A. baumannii* with CRISPRi. Lipoproteins are secreted from the cytosol and modified through several maturation steps by Lgt, LspA, and Lnt. Mature lipoproteins are extracted by LolDF and trafficked across periplasm by LolA. They are then transferred to LolB, the outer membrane acceptor that insert them into the OM. Unlike in *E. coli* and many other Gram-negative organisms, Lnt is not essential in *A. baumannii;* however deletion does result in increased OM permeability (12). CRISPRi gRNAs were designed to target *lgt* (A1S_0460), *lnt* (A1S_0373/4), and *lolA* (A1S_2729)*,* and these are denoted with red Xs.

The outer membrane (OM) is an essential protective barrier for Gram-negative bacteria, such as *Acinetobacter baumannii* (1). It is an asymmetric lipid bilayer containing phospholipids on the inner leaflet and lipopolysaccharide (LPS) or lipooligosaccharide (LOS) on the outer leaflet (2–4). There are four essential pathways that build the OM: Lpt, which transports LPS/LOS; Bam, which assembles β-barrel outer membrane proteins (OMPs) into the OM; a family of AsmA proteins likely transport phospholipids; and Lol, which traffics lipoproteins across the aqueous periplasm (1, 5–7). At least three of these pathways require one or more OM-localized lipoprotein components to function. Hence, the Lol pathway is pivotal for OM biogenesis. Lipoproteins destined for the OM first undergo several post-secretory maturation steps at the inner membrane (IM). First, the enzyme Lgt diacylates an invariant cysteine residue (termed Cys^+1^) (8). Signal peptidase LspA then cleaves the signal peptide sequence, allowing Cys^+1^ to become the N-terminus (9). Finally, N-acylation by Lnt adds a third and final acyl chain to the Cys^+1^ residue (10, 11). While all maturation steps are essential in *E. coli*, Lnt is not essential in *A. baumannii*, at least in laboratory conditions (12). Lipoproteins mature in the IM but most are destined for the OM, requiring trafficking across the aqueous periplasmic space. In *E. coli*, OM-targeted lipoproteins are extracted from the IM by the LolCD_2_E transporter (13, 14), the LolA periplasmic chaperone triggers their release and then traffics them to the OM where they are inserted into the bilayer by LolB (15–19). The trafficking pathway is thought to function analogously in *A. baumannii* (Fig. 1), although this organism produces a LolD_2_F_2_ IM transporter complex, where LolF resembles a hybrid of *E. coli* LolC and LolE (13). Since lipoprotein maturation is a prerequisite for trafficking to the OM, we refer to these sequential processes collectively as “OM lipoprotein biogenesis”.

Envelope stress responses (ESRs) ensure that integrity of the OM is maintained. ESRs are best understood in *E. coli* where distinct ESRs are tasked primarily with monitoring one OM component: LPS at the OM is primarily monitored by the Rcs stress response system (20, 21); OMP assembly by Bam is primarily monitored by the σ^E^ response (22–26). The Cpx stress response system monitors lipoprotein trafficking to the OM *via* Lol through the use of the OM-targeted lipoprotein NlpE (27–29). The Cpx response is a two-component system (TCS) consisting of sensor histidine kinase CpxA and response regulator CpxR (30, 31). When lipoprotein trafficking to the OM is defective, NlpE accumulates in the IM, and this mislocalization allows its N-terminal signaling domain to bind and activate CpxA, initiating the transcriptomic stress response *via* CpxR (27–29). While the OM biogenesis machines are broadly conserved throughout Gram-negative species, ESRs are not. There is little known about the ESRs that monitor OM assembly in *A. baumannii*. In fact, *A. baumannii* does not have a clear homolog of Rcs, σ^E^, or Cpx (26).

**Fig 2:**
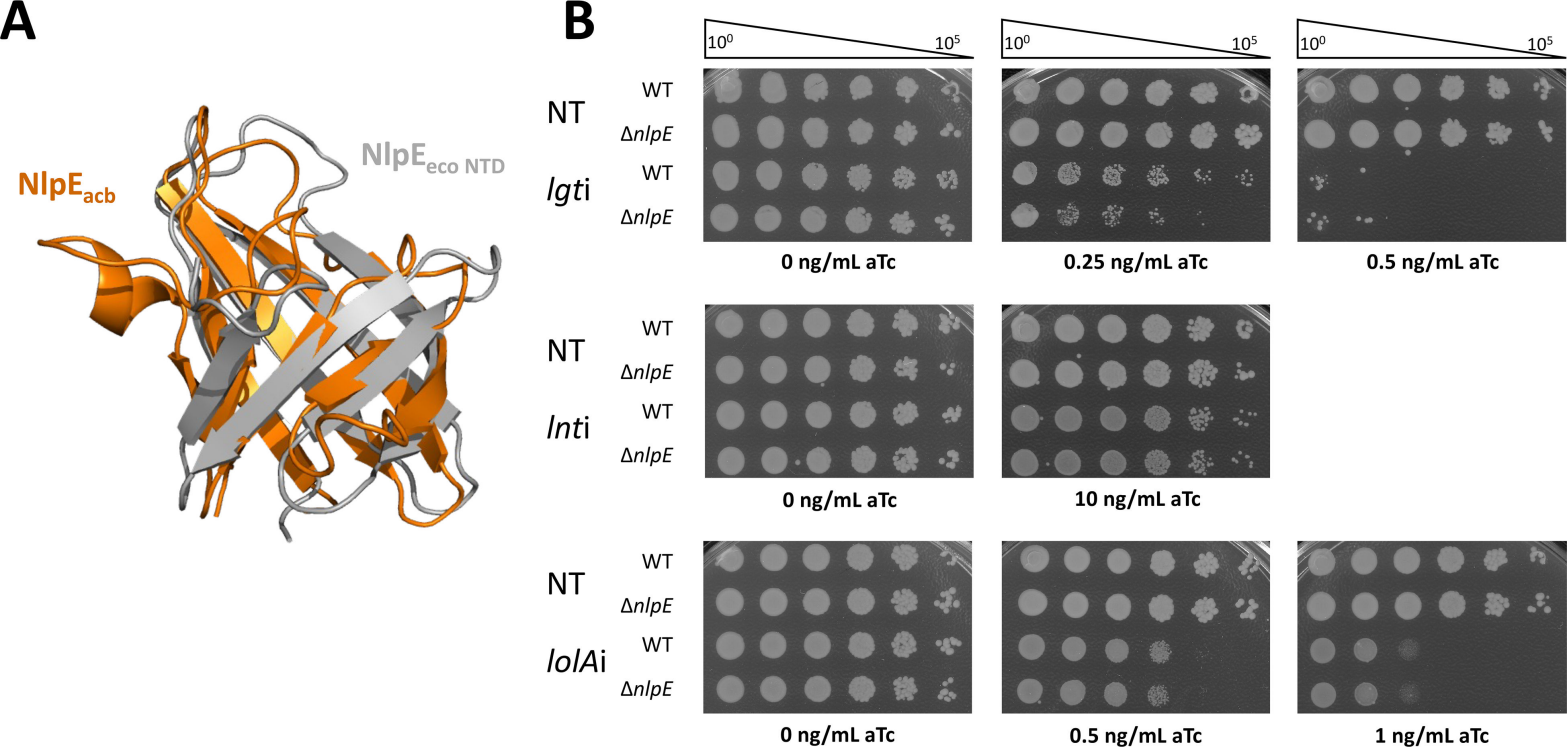
NlpE does not provide protection against lipoprotein trafficking defects in *A. baumannii*. (**A**) AlphaFold2 structural prediction of *A. baumannii* NlpE overlayed on *E. coli* NlpE_eco_ N-terminal domain (residues 1–121) crystal structure (PDB 2Z4H). *A. baumannii* NlpE inherently lacks a C-terminal domain that is present in *E. coli* NlpE but not depicted in this image (**B**) Wild-type and Δ*nlpE* (A1S_1009) mutant *A. baumannii* strains carrying either the non-targeting (NT), *lgt*, *lnt*, or *lolA* gRNA plasmid for CRISPRi depletion were grown overnight and plated in 10-fold serial dilution on LB + carbenicillin supplemented with 0.25–10 ng/mL aTc to induce dCas9 expression. Plates without aTc represent nondepleted control conditions. EOP are representative of biological triplicates.

We recently elucidated the mechanism of stress sensing by NlpE and its activation of Cpx in *E. coli* (28, 29). We sought to test if a comparable stress response mechanism exists in *A. baumannii* since this organism also encodes an NlpE with an N-terminal domain (NTD) that is homologous to the *E. coli* NlpE NTD that activates the CpxA sensor kinase. The function of *A. baumannii* NlpE is unclear; however, since Cpx is absent in this species (Fig. 2A) (32). To understand how *A. baumannii* withstands OM lipoprotein biogenesis stress and test whether NlpE is involved, we designed a CRISPRi approach to induce stress by depleting *lgt*, *lnt*, or *lolA* individually (Fig. 1). In this study, we defined the transcriptomic response of *A. baumannii* to OM lipoprotein biogenesis stress, identified, and measured changes in expression of all encoded lipoproteins, and discovered that *A. baumannii* combats lethal OM lipoprotein biogenesis stress using the BfmRS TCS through a novel stress sensing mechanism that does not rely on NlpE.

## RESULTS

### Deletion of *nlpE* does not sensitize *A. baumannii* to OM lipoprotein stress

An *E. coli* mutant lacking NlpE (Δ*nlpE*) is sensitized to defects in lipoprotein trafficking caused either by genetic depletion of essential steps or by chemical inhibition of essential steps (27–29). *E. coli* NlpE protects against such defects by activating the Cpx stress response (27–29). Although *A. baumannii* lacks the Cpx system, it does encode a *nlpE* homolog (A1S_1009) that is smaller than *E. coli* NlpE (*A. baumannii* NlpE lacks a globular C-terminal domain) but retains an NTD that shares 30% amino acid identity and structural similarity with the NTD of *E. coli* NlpE that activates Cpx (Fig. 2A). We explored whether NlpE function in mitigating lipoprotein trafficking stress is conserved between *E. coli* and *A. baumannii*. We induced stress in either lipoprotein maturation or trafficking by using CRISPRi genetic depletion of essential proteins both in wild-type (*nlpE*^+^) and isogenic Δ*nlpE::kan A. baumannii* ATCC 17978 strains.

We adapted a CRISPRi system previously developed in *A. baumannii* ATCC 17978 (33) that comprises an anhydrotetracycline (aTc)-inducible dCas9 gene inserted at single copy in the chromosomal *att*Tn7 site and a constitutively expressed gRNA *via* high-copy-number plasmid pYDE007 (33). gRNAs were designed to target discreet steps of OM lipoprotein maturation and trafficking: *lgt* (A1S_0460), *lnt* (A1S_0373/4), and *lolA* (A1S_2729) (Fig. 1). A non-targeting (NT) gRNA (designed to target *mrfp*) was used as a negative control, as previously described (33). gRNA-expressing plasmids were introduced into *A. baumannii att*Tn7::dCas9 strains with either *nlpE^+^* or Δ*nlpE::kan*. In our systems, transcription of *lgt*, *lnt*, or *lolA* is inhibited when culturing with aTc to induce dCas9 (such treatment is abbreviated as *lgti*, *lnti*, and *lolAi* herein), and hence levels of the encoded protein are depleted over time.

Viability of each depletion was evaluated by an efficiency of plating (EOP) assay. Strains were plated in a serial 10-fold dilution on LB agar plates supplemented with varying concentrations of aTc inducer, optimized for each gRNA target. The more inducer, the more depletion of the target. We found that the wild-type and Δ*nlpE::kan* strains had a comparable and dose-dependent reduction in viability during *lgti* or *lolAi* (Fig. 2B). As expected, *lnti* was not lethal since complete deletion of *lnt* is tolerated in wild-type *A. baumannii* (12); the loss of *nlpE* apparently did not alter the non-essentiality of *lnt* (Fig. 2B) (12). Overall, unlike in *E. coli*, deletion of *nlpE* did not sensitize *A. baumannii* to induce defects in OM lipoprotein maturation (*lgti*) or trafficking (*lolAi*). These data suggested that *A. baumannii* NlpE is likely not involved in sensing and protecting against defects in OM lipoprotein biogenesis (28).

### The transcriptomic response to defects in OM lipoprotein biogenesis

Since *A. baumannii* lacks a Cpx homolog, and our data suggested that NlpE does not participate in protecting the cell against OM lipoprotein trafficking defects, we turned to an unbiased approach to define the transcriptional response of *A. baumannii* facing CRISPRi-induced defects in OM lipoprotein biogenesis. RNA was isolated from *lgti*, *lnti*, and NT control treated strains in otherwise wild-type *A. baumannii* ATCC 17978 to assess gene expression changes in response to OM lipoprotein biogenesis stress. RNA-Seq analysis was used to quantitatively measure the abundance of each gene’s transcript.

**Fig 3:**
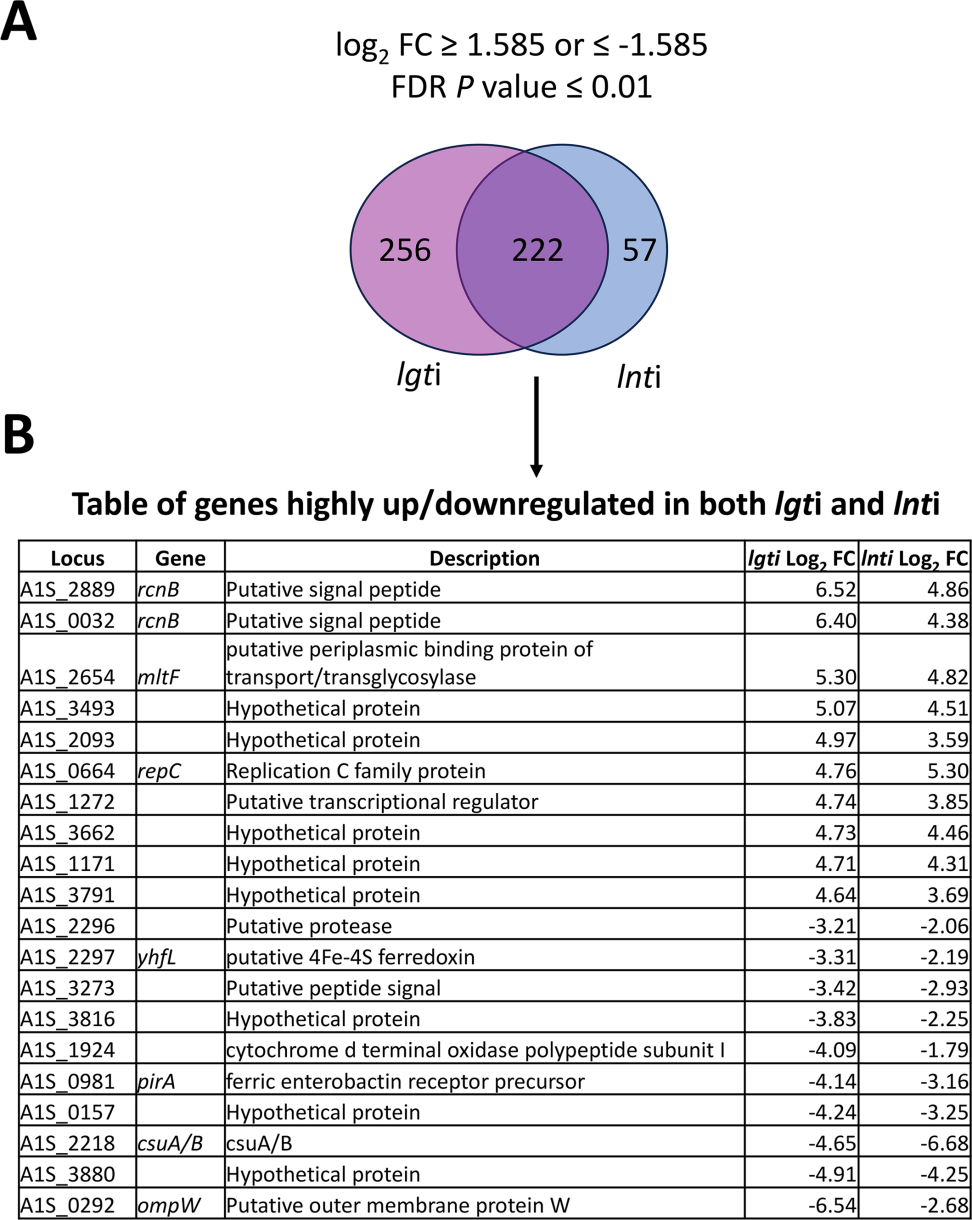
Genes significantly upregulated and downregulated under OM lipoprotein biogenesis stress. (**A**) RNA-seq was performed using RNA isolated from *A. baumannii* CRISPRi strains carrying non-targeting, *lgt* targeting (*lgti*), or *lnt* targeting (*lnti*) gRNA plasmids. Gene depletions were induced with aTc, and cells were isolated at mid-log. Log_2_FC values were calculated by comparing *lgti* and *lnti* strains to non-targeting control, *n* = 2 per strain. The log_2_FC cutoff used was ±1.585 for ~3 FC up- or downregulated under depletion compared with the control with significant FDR *P*-values < 0.01. The Venn diagram represents the number of significant hits that fall in the set threshold that is unique to *lgti* or *lnti* or shared between the data sets (*lgti* and *lnti*). (**B**) Log_2_FC for ten most up/downregulated genes shared between the *lgti* and *lnti* data sets is listed in the order of most upregulated to most downregulated.

We identified many more differentially regulated genes during *lgti* than *lnti*, likely reflecting the fact that *lgti* is ultimately a lethal treatment while *lnti* is not (Fig. 3A). Crucially, we identified a set of genes that exhibited the same differential regulation during both *lgti* and *lnti* treatments (Fig. 3B). Our RNA-Seq analysis offers the first transcriptomic signature for the response of *A. baumannii* to OM lipoprotein biogenesis stress (Fig. 3B; Table 1; Table S1). Fold changes in mRNA levels in *lgti* or *lnti* were calculated by comparing reads to the NT control treatment. CRISPRi-targeted depletion of *lgt* yielded downregulation of −5.57 log_2_FC (Table 1), and depletion of *lnt* yielded downregulation of −4.48 log_2_FC (Table 1), supporting that the gRNAs knocked-down the intended target genes. Additionally, *lolA* and *lolB* lipoprotein trafficking genes were upregulated under CRISPRi-induced trafficking stress (Table 1). Under *lgti*, *lolA* was upregulated 2.97 log_2_FC, and *lolB* was upregulated 2.09 log_2_FC (Table 1). Since *lnt* is not essential, we expected that stress induced by *lnti* would not be as severe as *lgti* (which is ultimately a lethal stress). Under *lnti*, *lolA* was upregulated 1.45 log_2_FC and *lolB* was not significantly upregulated.

**TABLE 1:** Genes regulated under OM lipoprotein biogenesis stress identified in other previously described in *Acinetobacter baumannii* stress responses^*a*^

Locus	Gene	Description	*Lgti* Log_2_ FC	*Lnti* Log_2_ FC
*Lipoprotein maturation and trafficking*				
A1S_0460	*lgt*	Prolipoprotein diacylglycerol transferase	−5.57	ns
A1S_0374	*lnt*	Apolipoprotein N-acetyltransferase	ns	−4.48
A1S_2729	*lolA*	Outer membrane lipoprotein carrier	2.97	1.45
A1S_0835	*lolB*	Outer membrane lipoprotein insertase	2.09	ns
*Stress response systems*				
A1S_1393	*amsS*	Hybrid sensor histidine kinase	2.3	2.05
A1S_1394	*amsR*	Response regulator	2.07	ns
A1S_2884	*baeS*	Sensor histidine kinase	1.41	ns
*Bae regulon*				
A1S_0538	*macA*	Efflux pump subunit	2.94	1.76
A1S_0536	*macB*	Efflux pump subunit	2.5	1.79
A1S_0535	*tolC*	Efflux pump subunit	2.3	1.65
A1S_1750	*adeB*	Efflux pump subunit	1.51	1.96
A1S_1751	*adeA*	Efflux pump subunit	ns	1.88
A1S_1769		tolC like subunit	2.66	2.52
A1S_2218	*csuA/B*		−4.65	−6.68
*Bfm regulon*				
A1S_2885		Putative lipoprotein	4.04	2.72
A1S_1193	*ompA/motB*	Outer membrane protein	1.53	1.67
A1S_2889		Hypothetical	6.52	4.86
A1S_2325		*ompW* like protein	4.04	3.1
A1S_3791		Putative OM barrier functional protein	4.64	3.69
A1S_3253		Hypothetical	1.75	2.11
A1S_0032		Hypothetical	6.4	4.38
A1S_3317		Outer membrane protein	1.81	1.51
A1S_1377		TetR transcriptional regulator	1.92	1.35
*Association with OM stress (LOS deficiency*)				
A1S_2654	*mltF*	Periplasmic binding protein of transport/transglycosylase	5.3	4.82
A1S_0292	*ompW*	Putative outer membrane protein W	−6.54	−2.68
A1S_3493		Lipoprotein	5.07	4.51
A1S_2157		Hypothetical	3.19	2.31
A1S_3127		Hypothetical	2.16	1.86
A1S_3034		Hypothetical	2.49	1.25
A1S_0158		Lipoprotein	2.61	ns

^*a*^ Genes were previously identified to be associated with BaeSR system (34–36), BfmRS system (37), and LOS deficiency (38). Log_2_FC for genes of interest from RNA-seq listed, all values have FDR *P*-values < 0.05. Genes with nonsignificant values are denoted with “ns”. Fold change calculated by comparing *lgti* and *lnti* strains to non-targeting control, *n* = 2 per strain.

**Fig 4:**
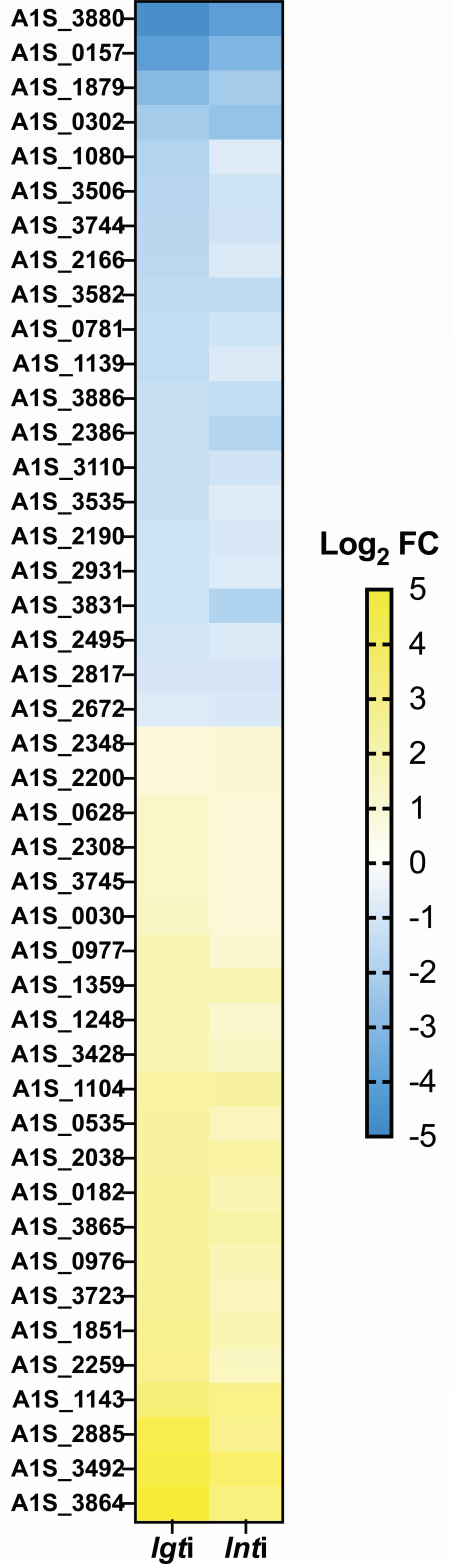
OM lipoprotein biogenesis stress impacts regulation of 44 lipoproteins. A signal P algorithm was used to predict lipoproteins in entire *Acinetobacter baumannii* ATCC 17978 genome. Out of the 130 predicted lipoproteins, 44 had significant upregulation or downregulation in response to lipoprotein trafficking stress (*lgti* and *lnti* RNA-seq, *P* < 0.05). All log_2_FC values are reported where yellow (>0) indicates upregulation and blue indicates downregulation (<0).

A feature of some ESRs is downregulation of genes encoding OM components when their transport or insertion into the OM is defective. To identify the effect of *lgti* and *lnti* on the transcription of lipoprotein genes, we first used the SignalP algorithm to globally predict all likely lipoproteins in *A. baumannii* ATCC 17978. SignalP prediction is based on identifying the conserved lipobox feature, which is required for lipoprotein secretion and maturation. Our data set identified 130 total likely lipoproteins (Table S2), and we found that 44 of these were significantly and consistently up- or downregulated during both *lgti* and *lnti* (Fig. 4). These gene expression changes potentially reflect prioritization of lipoprotein synthesis during stress in the OM lipoprotein biogenesis pathway.

By assessing changes in expression of genes encoding elements of signal transduction systems (*e.g.*, TCS), we identified two candidate stress response systems, *amsSR* (A1S_1393 and A1S_1394) and *baeSR* (A1S_2884 and A1S_2883), that were significantly upregulated in *lgti* (Table 1). We hypothesized that either (i) these systems might be involved in sensing and combatting OM lipoprotein biogenesis stress or alternatively, (ii) they may simply be activated as a consequence of stress caused by defective OM lipoprotein biogenesis.

### The contributions of AmsSR and BaeSR TCSs during *lgti* and *lnti* depletions

**Fig 5:**
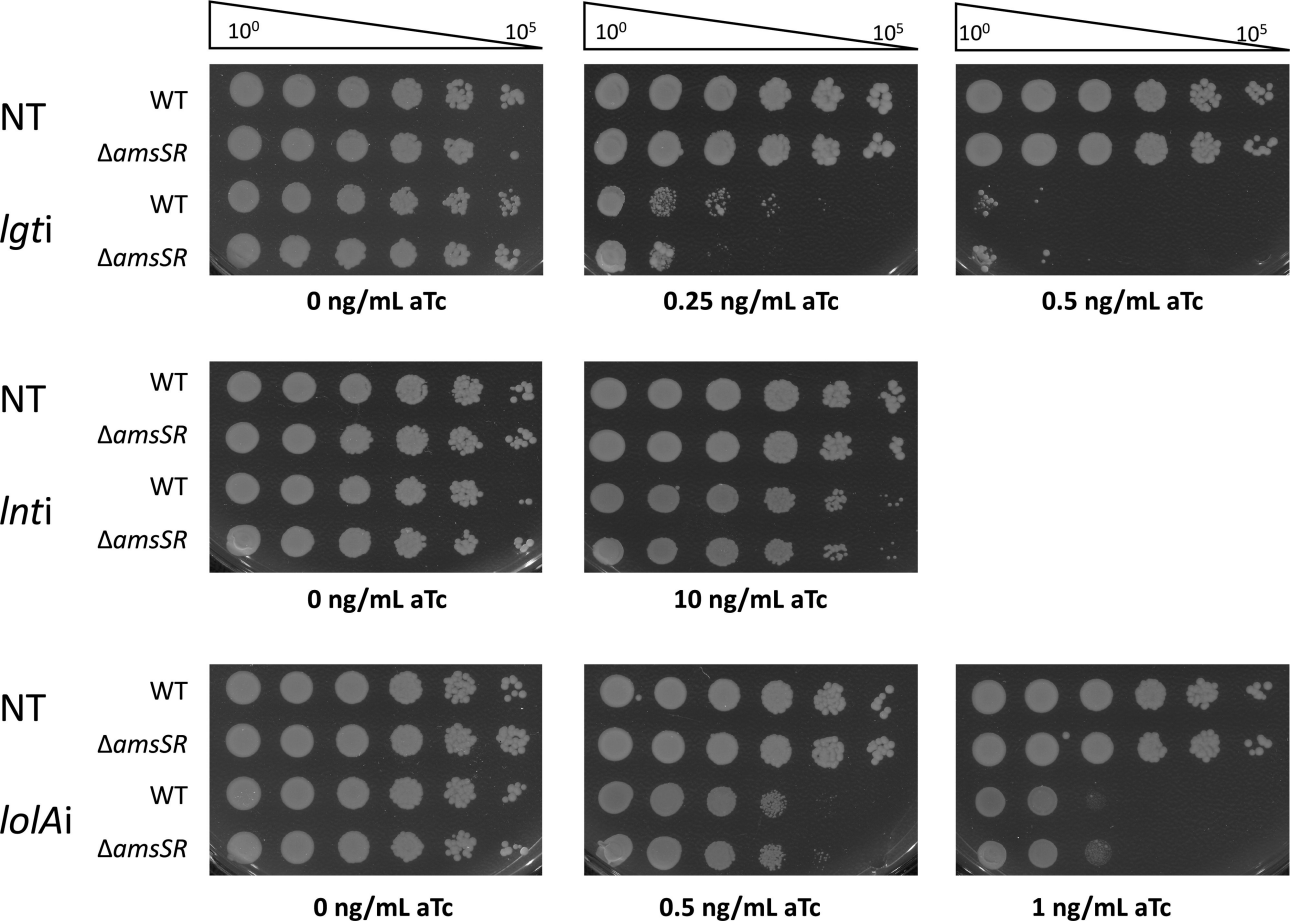
AmsSR is not involved in monitoring OM lipoprotein biogenesis. Wild-type and *amsSR* mutant strains carrying either the non-targeting (NT), *lgt*, *lnt*, or *lolA* sgRNA plasmid for CRISPRi depletion were grown overnight and plated in 10-fold serial dilution on LB + carbenicillin supplemented with 0.25–10 ng/mL aTc to induce dCas9 expression. Plates without aTc represent nondepleted conditions. EOP are representative of biological triplicates.

The AmsSR system is a recently identified atypical two-component system (39). AmsS is a hybrid histidine kinase with an unusual number of transmembrane domains, a conserved histidine residue that is autophosphorylated by the ATPase domain, and a response regulator receiving domain with a conserved aspartate residue to receive the phosphate (39, 40). This system also has an additional response regulator with a receiving domain and DNA binding domain (41). One described function for AmsSR suggests it regulates metabolic pathways favored in limited oxygen environments (39). We constructed an *amsSR* null mutation (Δ*amsSR*) into the *att*Tn7::dCas9 strain by recombineering and then introduced gRNA-expressing plasmids targeting *lgt*, *lnt*, or *lolA* in order to induce OM lipoprotein biogenesis stress. Viability of Δ*amsSR* under lipoprotein trafficking stress was assessed through EOP on plates containing a range of aTc concentrations. Deletion of *amsSR* had no impact on viability under any of the depletion conditions, as mutant strains appeared just as sensitive as wild-type across several aTc concentrations (Fig. 5). These data suggested that AmsSR does not play a role in protecting against OM lipoprotein trafficking stress. Rather, AmsSR might be activated during OM lipoprotein trafficking stress as a consequence of ensuing cell envelope defects.

**Fig 6:**
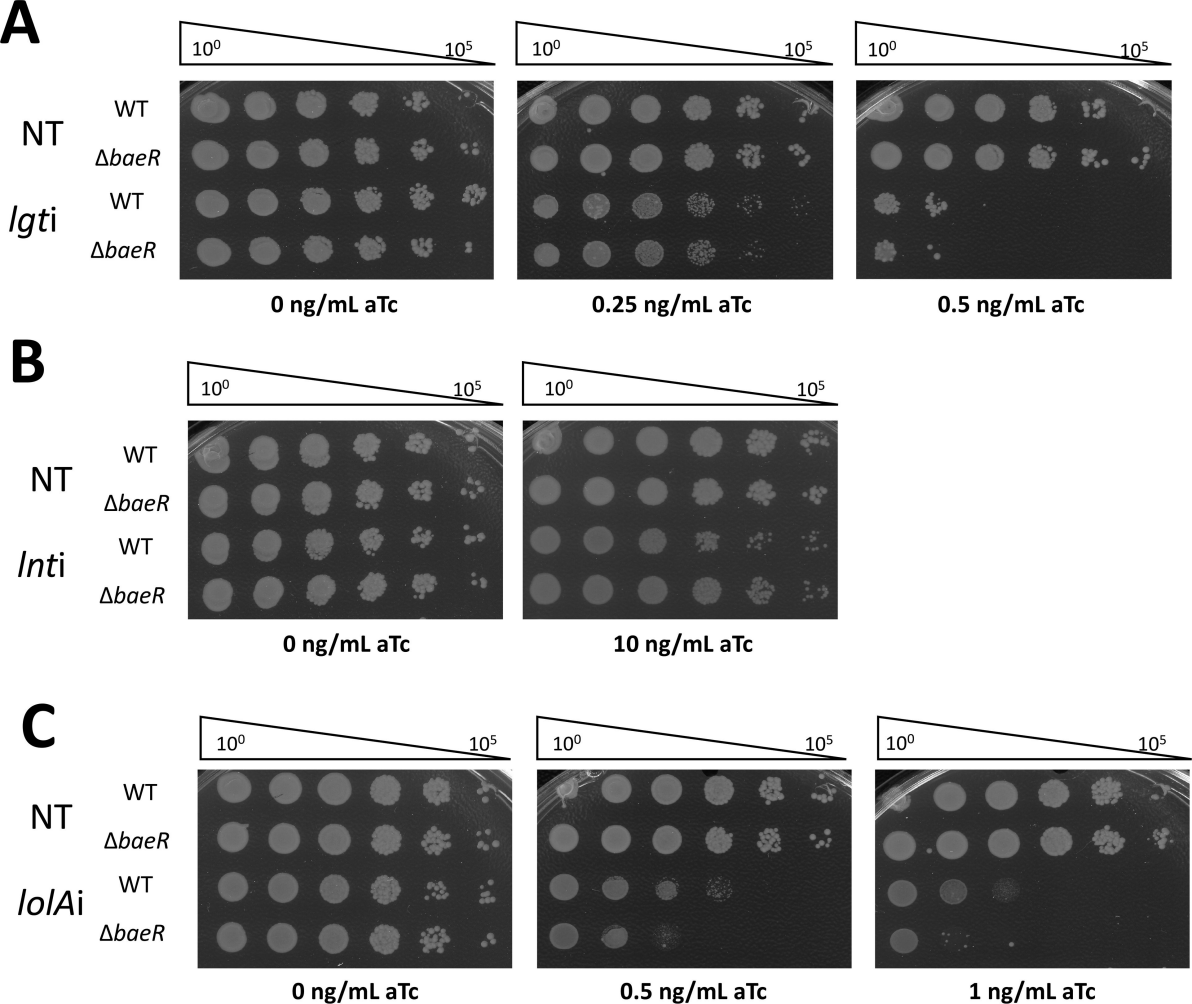
BaeSR does not provide protection against defects in OM lipoprotein biogenesis. Wild-type and *baeR* mutant strains carrying either the non-targeting (NT), *lgt*, *lnt*, or *lolA* sgRNA plasmid for CRISPRi depletion were grown overnight and plated in 10-fold serial dilution on LB + carbenicillin supplemented with 0.25–10 ng/mL aTc to induce dCas9 expression. Biological triplicates shown are representative of the variability seen in depletion sensitivity.

The *A. baumannii* BaeSR system, similarly to its homolog in *E. coli*, is a TCS that regulates efflux pumps and responds to high osmolarity and toxic compounds (such as antibiotic therapy) (34–36). BaeS is the sensor histidine kinase and BaeR is the response regulator (42). We constructed a *baeR* null mutation (Δ*baeR*) in the *att*Tn7::dCas9 strain using recombineering and introduced gRNA-expressing plasmids targeting for *lgt*, *lnt*, or *lolA*. Viability of the Δ*baeR* mutant under lipoprotein trafficking stress was assessed through EOP on plates containing a range of aTc concentrations. Deletion of *baeR* had no impact on viability under *lgti* or *lnti*, since Δ*baeR* appeared just as sensitive as wild-type across several aTc concentrations (Fig. 6A and B). Interestingly, the Δ*baeR* strain was clearly more sensitive to *lolAi* than wild-type (Fig. 6C).

### BaeR promotes expression of *lolA*

Upregulation of the Lol trafficking pathway (most prominently *lolA*) had been previously reported in *A. baumannii* strains that lacked LOS, a severe cell envelope disruption. Transcriptomics of LOS deficient strains revealed that the BaeSR system is also upregulated in LOS-deficient cells (38, 43). Indeed, transcriptomes of LOS-deficient cells and *lgti* and *lnti A. baumannii* cells share a common signature of strongly upregulating *lolA* and *tolC* (A1S_0535) while strongly downregulating *ompW* (A1S_0292) (Table 1).

**Fig 7:**
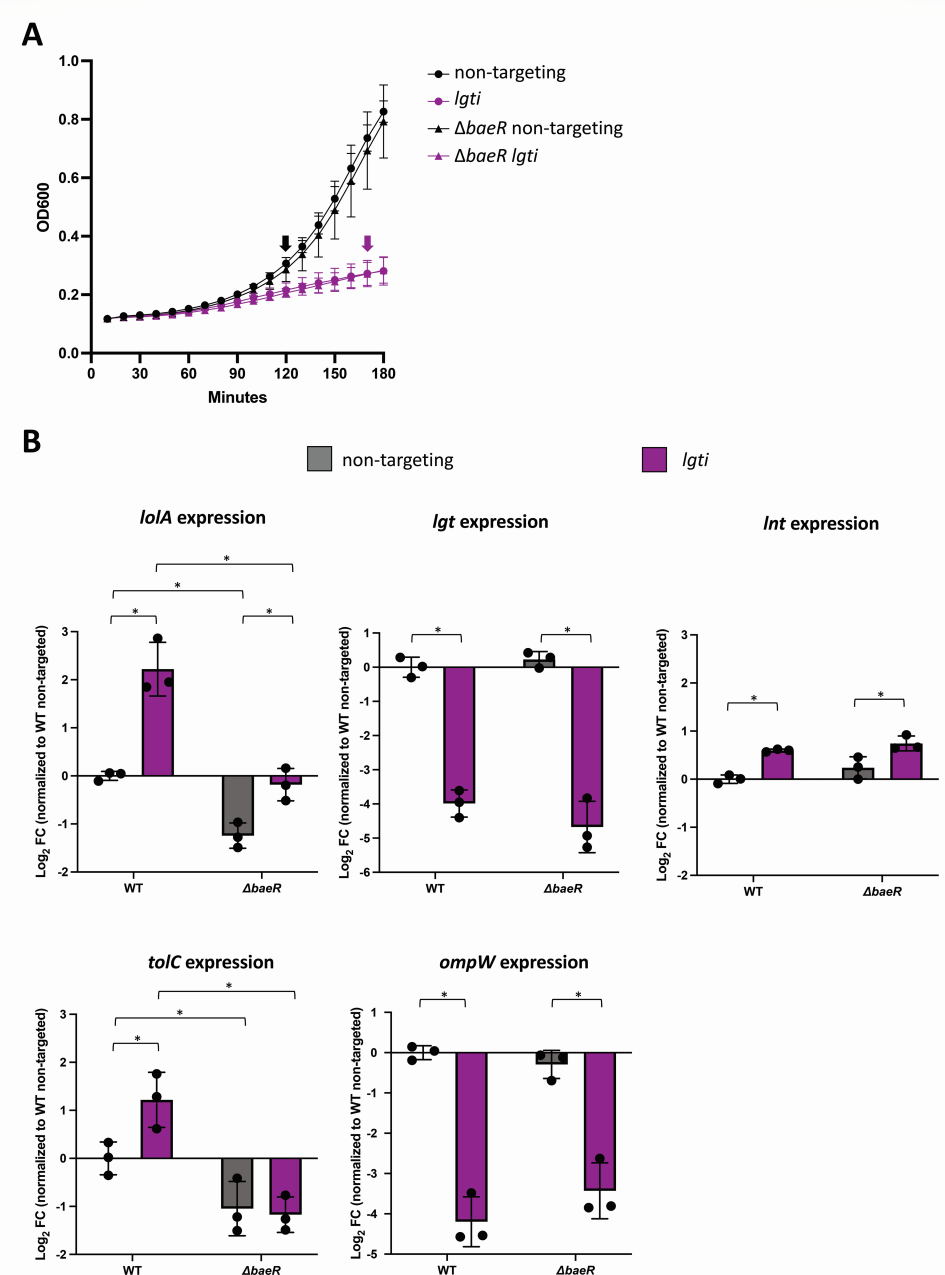
BaeR regulates *lolA* expression. **A**) RNA was isolated from *lgt* deplete cells or non-targeting control in wild-type and *baeR* mutants from three biological replicates; cells sampled denoted by arrow. **B**) Expression of *lolA* (A1S_2729), *lgt* (A1S_0460), *lnt* (A1S_0373/4), *tolC* (A1S_0535), and *ompW* (A1S_0292) and was measured *via* RT-qPCR. Log_2_FC was calculated *via* the ΔΔCT method, and expression was compared to the WT strain under non-targeting conditions. A two-way ANOVA was performed to compare normalized expression. * represents *P* < 0.05, ** represents *P* < 0.01, *** represents *P* < 0.001, **** represents *P* < 0.0001. Gray bars represent non-targeting depletion and purple *lgt* depletion.

Our CRISPRi data showed that Δ*baeR* is as robust as wild type to depletions targeting early steps in OM lipoprotein biogenesis (*lgti* and *lnti*) but, surprisingly, is sensitive to depletion in the late LolA step (*lolAi*). Those findings argued against Bae being a general stress response for OM lipoprotein biogenesis. Rather, the data suggested a limited role for BaeR in protecting *A. baumannii* specifically against LolA depletion. We hypothesized that BaeR may regulate *lolA* to promote its expression; hence, Δ*baeR* sensitivity to *lolAi* may reflect the reduced basal level of *lolA* transcription in the mutant. To test this hypothesis, we performed RT-qPCR of wild-type and Δ*baeR* cells, examining mRNA levels of *lolA* as well as *tolC* and *ompW* that appear to correlate with Bae upregulation during both LOS deficiency and OM lipoprotein biogenesis stress. RNA was isolated from wild-type and Δ*baeR* strains, carrying either non-targeting control (NT) or *lgt* gRNA plasmid, grown to log-phase in LB supplemented with 25 ng/mL aTc (Fig. 7A). In the absence of OM lipoprotein biogenesis stress (NT control), the amount of *lolA* mRNA is significantly reduced by −1 log_2_FC in Δ*baeR* compared with wild type (Fig. 7B), suggesting that BaeR is a regulatory input for basal *lolA* expression in *A. baumannii*. Under *lgti* conditions, the Δ*baeR* strain also produced significantly less *lolA* mRNA than wild-type *lgti*-treated cells (Fig. 7B). Expression of other OM lipoprotein biogenesis genes *lgt* and *lnt* was unchanged between Δ*baeR* and wild type (Fig. 7B). Notably, we still observed an *lgti* stress-dependent induction of *lolA* in Δ*baeR*, suggesting that stress activation of *lolA* expression is BaeR-independent (Fig. 7B). Hence, RT-qPCR analysis supported our hypothesis that the specific sensitivity of Δ*baeR* to only *lolAi* (and not *lgti* or *lnti*) is due to inherently lower *lolA* expression in Δ*baeR* than wild type, which likely enhances gene depletion when CRISPRi targeting *lolA* is induced.

Expression of *tolC*, an OM pore component of antibiotic efflux pumps, was also significantly reduced in the Δ*baeR* strain in NT conditions (Fig. 7B). Expression of *tolC* was induced by *lgti* conditions, and this increase was fully BaeR-dependent since Δ*baeR* expressed *tolC* at equivalent levels in both unstressed NT conditions and stressed *lgti* conditions (Fig. 7B). These data confirmed that *tolC* is a part of the known Bae regulon (34). While downregulation of *ompW* occurred concomitantly with *baeR* upregulation in our RNA-Seq of OM lipoprotein biogenesis stress (and was also seen in LOS-deficient strains (Table 1 and [38]), we found that BaeR was not involved in *ompW* regulation. Levels of *ompW* transcript were similarly decreased during *lgti* in both Δ*baeR* and wild-type cells (Fig. 7B).

### The BfmRS response is crucial for mitigating OM lipoprotein biogenesis stress

**Fig 8:**
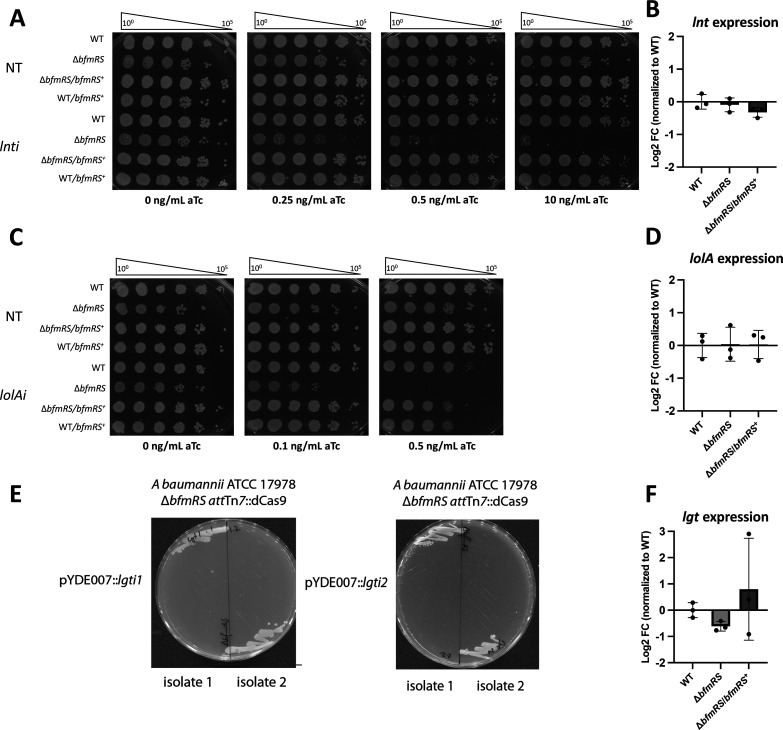
BfmRS is critical for responding to lipoprotein trafficking defects. (**A and C**) Wild-type and *bfmRS* mutant strains carrying either the non-targeting (NT), *lgt*, *lnt*, or *lolA* sgRNA plasmid for CRISPRi depletion were grown overnight and plated in 10-fold serial dilution on LB + carbenicillin supplemented with 0.1–10 ng/mL aTc to induce dCas9 expression. Plates are representative of biological triplicates. (B, D, and F) RNA was isolated from wild-type, *bfmRS,* and complement strains from three biological replicates at midlog phase. Expression of *lgt* (A1S_0460), *lnt* (A1S_0373/4), and *lolA* (A1S_2729) was measured *via* RT-qPCR. Log_2_FC was calculated *via* the ΔΔCT method, and expression was compared with the WT strain. A two-way ANOVA was performed to compare normalized expression and asterisk (*) represents *P* < 0.05. (**E**) Initial colonies following transformation with either *lgti1* or *lgti2* gRNA plasmids were isolated on LB + carbenicillin and then streaked onto LB + carbenicillin plates. Representative streaks demonstrate that transformants failed to grow on these media.

The BfmRS TCS was suggested to regulate transcription of a number of genes involved in OM and peptidoglycan cell wall assembly and maintenance, including lipoprotein maturation enzymes *lgt* and *lnt* as well as the entire Lol pathway (*lolF*, *lolD*, *lolA*, and *lolB*) (37). Surprisingly, given its proposed central role in regulating OM and cell envelope biogenesis pathways, *bfmRS* was not previously been found to be upregulated during severe genetic disruption to the cell envelope such as LOS deficiency (38, 43). Our RNA-Seq data also did not suggest a transcriptional increase in *bfmR* or *bfmS* following either *lgti* or *lnti*, and we additionally confirmed that no change in *bfmR* expression occurred during *lgti* by RT-qPCR (Fig. S2). Nonetheless, we tested whether the Bfm TCS may be involved in combatting OM lipoprotein biogenesis stress. In this system, BfmS is the histidine kinase, and BfmR is the response regulator (44–47). gRNA-expressing plasmids targeted for *lnt* and *lolA* were introduced into Δ*bfmRS*, and viability was assessed by EOP in wild-type and Δ*bfmRS* strains. While *lolA* depletion was well tolerated when dCas9 was induced with up to 1 ng/mL aTc in a wild-type background was well tolerated, the Δ*bfmRS* mutant was not viable at aTc levels down to 0.25 ng/mL (Fig. 8C). Hence, Δ*bfmRS* cells more acutely lost viability during *lolAi*, similar to how loss of the Cpx response more acutely sensitized *E. coli* to depletion of OM lipoprotein trafficking. While, *lnti* was non-lethal in wild-type *A. baumannii* (where *lnt* deletion is also tolerated), the Δ*bfmRS* mutant was surprisingly highly sensitive to *lnti* (Fig. 8A); this finding suggested that the Bfm stress response is ordinarily required by wild-type *A. baumannii* to survive Lnt-deficiency. The Δ*bfmRS* mutant also appeared to be highly sensitive to *lgti*: while we could initially isolate transformants carrying gRNA plasmid targeting *lgt*, these transformants proved to be ultimately non-viable (Fig. 8E). We also generated a second gRNA plasmid targeting *lgt* at a different sequence of the gene and observed the same non-viable phenotype when introducing the plasmid into Δ*bfmRS att*Tn7::dCas9. Those findings strongly indicated that CRISPRi targeting *lgt* is lethal to Δ*bfmRS* even at basal dCas9 expression levels (*i.e.*, even without aTc induction). Importantly, we were able to complement the sensitivity Δ*bfmRS* to *lnti* and *lolAi* when we introduced *bfmRS* into an ectopic chromosomal locus (*hisG*). Complementation restored tolerance to lipoprotein trafficking stress comparable to the wild-type strain, further supporting that the BfmRS system is important for survival under lipoprotein trafficking stress (Fig. 8A and C).

The sensitivity of Δ*bfmRS* to *lgti, lnti,* or *lolAi* was not due to reduced expression of these genes in the Δ*bfmRS* mutant (that could have enhanced CRISPRi depletion). We measured expression of each gene in wild-type, Δ*bfmRS,* and Δ*bfmRS hisG::bfmRS* strains by RT-qPCR and detected comparable expression (Fig. 8F, B and D). These gene expression data argued that Δ*bfmRS* sensitization to *lgti*, *lnti*, and *lolAi* was due to the absence of an adaptive response. We also found that BfmRS is not responsible for hallmark *ompW* downregulation or *tolC* upregulation (Fig. S1).

**Fig 9:**
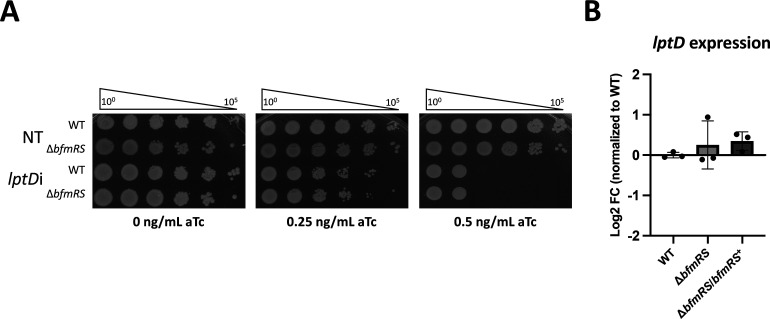
BfmRS mutant is not sensitized by LOS depletion (*lptDi*). **A**) Wild-type and *bfmRS* mutant strains carrying either the non-targeting (NT) or *lptD* sgRNA plasmid for CRISPRi depletion were grown overnight and plated in 10-fold serial dilution on LB + carbenicillin supplemented with 0.25–0.5 ng/mL aTc to induce dCas9 expression. Plates are representative of biological triplicates. **B**) RNA was isolated from wild-type, *bfmRS,* and complement strains from three biological replicates at midlog phase. Expression of *lptD* (A1S_1546) was measured *via* RT-qPCR. Log_2_FC was calculated *via* the ΔΔCT method, and expression was compared with the WT strain. A two-way ANOVA was performed to compare normalized expression and asterisk (*) represents *P* < 0.05.

The deletion of *bfmRS* highly sensitized *A. baumannii* to any defect in OM lipoprotein biogenesis. We wondered whether this effect was specific to the lipoprotein pathway or if it reflected a generalized sensitivity to OM or cell envelope perturbation. To test between these possibilities, we induced defects in the Lpt system that is also essential for OM assembly but is responsible for LOS transport to the OM. We targeted *lptD* for CRISPRi depletion; this gene encodes the final step in the LOS transport pathway. As expected, *lptDi* reduced cell viability in an aTc-dependent manner. Notably, viability of wild-type and Δ*bfmRS* strains under *lptDi* was comparable (Fig. 8C); expression of *lptD* was unchanged in the Δ*bfmRS* strain compared with wild type (Fig. 9B). Our data suggested that BfmRS is crucial for preserving viability specifically under OM lipoprotein biogenesis stress and not under other stress occurring in another essential OM assembly pathway (Fig. 8 and 9).

## DISCUSSION

The OM of Gram-negative bacteria is an essential structure that forms the interface between the bacteria and their host or environment. In human pathogens, such as *A. baumannii*, the OM is likely under continuous assault from innate immune effectors, environmental stressors, and antibiotic therapy. The OM provides a highly impermeable barrier to prevent such toxins from killing the cell (48). It is critical for *A. baumannii* survival to ensure that OM integrity is continuously maintained. Construction of the OM is achieved through an orchestrated synthesis, transport, and assembly of the lipid and protein building blocks (49–52). Since OM assembly processes are inter-dependent, a defect in one process can propagate across multiple processes (50). ESRs have evolved to constantly monitor the fidelity of OM assembly pathways and integrity of the OM itself (53). Decades of study defined the *E. coli* ESRs and delineated how each ESR is typically tasked primarily with monitoring a discreet OM component and its assembly into the membrane (53). However, these ESRs are not widely conserved. As a result, there is limited understanding of how distantly related pathogens, such as *A. baumannii*, ensure their OM integrity. ESRs are well-known contributors to the intrinsic antibiotic resistance of *E. coli* (54); understanding ESRs in *A. baumannii* will likely similarly inform how these bacteria protect themselves against antibiotic therapy, a key goal given the broad antibiotic-resistance that is frequent among clinical *A. baumannii* isolates.

In this study, we focused on OM lipoprotein biogenesis—encompassing both the acylation maturation steps and the Lol trafficking system. OM lipoproteins are central to building the OM since they play essential roles in each of the OM assembly pathways (Bam, Lpt, Lol) (50); indeed, OM lipoprotein biogenesis has proven to be an attractive target for novel antibiotics targeting *A. baumannii* (55–58). Defects in delivering lipoproteins to the OM would cause wholesale defects throughout OM assembly processes. We reasoned, then, that it is highly likely that *A. baumannii* would monitor this pathway to mitigate the consequences of any defects in OM lipoprotein biogenesis.

ESR reporters and transcriptional profiling are useful tools in the quest to discover or define targets for novel antibacterials. Our study used CRISPRi to induce defects in OM lipoprotein biogenesis. Our transcriptomic analysis offers the first snapshot of the *A. baumannii* response to OM lipoprotein biogenesis defects. We used RNA-Seq to characterize the response to CRISPRi of two independent targets in OM lipoprotein biogenesis to cause a stress that (i) ultimately leads to cell death (*lgti*) and (ii) is tolerated and non-lethal (*lnti*). We found that *tolC* and *ompW* are strongly up- and downregulated, respectively; such expression of these two genes was also detected in LOS deficient *A. baumannii*, suggesting they are robust hallmarks of severe OM disruption (38). Our data show that BaeR is responsible for the *tolC* up-regulation, but the mechanism for decreased *ompW* mRNA levels is still not clear. LOS-deficient *A. baumannii* ATCC 17978 was also previously found to have elevated levels of both *lolA* and *baeSR* mRNA (38). Our data demonstrate that BaeR participates in basal expression of *lolA*. Levels of *lolA* mRNA levels are reduced in Δ*baeR* but remain inducible in response to *lgti* stress. The source of stress-induced *lolA* up-regulation is not clear, although our data exclude BfmR. Our Δ*baeR* mutant exhibited increased sensitivity to *lolAi* but not to either *lgti* or *lnti*. The simplest model to explain why Δ*baeR* mutants were more sensitive specifically to *lolAi* is that the lowered basal expression of *lolA* in this strain simply causes more efficient depletion once dCas9 is induced in *lolAi*. The fact that Δ*baeR* mutants are not more sensitive to *lgti* or *lnti* than wild type argues against a generalized role for Bae in responding to OM lipoprotein biogenesis stress. In contrast, Δ*bfmRS* mutants are sensitive to CRISPRi depletion of each step in OM lipoprotein biogenesis that we targeted, a hallmark of a dedicated ESR. It may seem odd that Bae regulates *lolA* but does not appear to protect cells against OM lipoprotein biogenesis stress. However, the *E. coli* Rcs system is wired analogously: Rcs upregulates *lolA* expression (59), but the Rcs response is not helpful during OM lipoprotein biogenesis stress (60). In fact, Rcs upregulates an OM lipoprotein, OsmB, that is toxic when mislocalized and Rcs activation actually impairs viability to severe OM lipoprotein biogenesis stress (60). The analogy to Rcs offers a possible hypothesis for why Bae regulates LolA—its regulon may contain an OM-targeted lipoprotein that, like OsmB, needs to be promptly removed from the IM to avoid toxicity to the cell.

Our data clearly demonstrate that BfmRS is tasked with mitigating OM lipoprotein biogenesis stress to preserve *A. baumannii* viability. Not only is BfmRS needed to preserve cell viability during CRISPRi depletion the essential genes *lgt* and *lolA*, but the loss of *bfmRS* severely impairs cell viability during depletion of non-essential *lnt*. The BfmRS system regulates many of the genes that are typically regulated by the RpoS stationary phase response in *E. coli* and has been proposed as a potential generalized stress response system (46, 61). However, in its capacity as an ESR, BfmRS appears to be specific for the OM lipoprotein biogenesis pathway, since it fails to offer protection against CRISPRi *lptD* depletion that deprives the OM of LOS. How does the BfmRS response protect the cell against the consequence of poor OM lipoprotein biogenesis? We do not detect changes in expression of *lgt*, *lnt*, or *lolA* when *bfmRS* is inactivated, suggesting that protection is not simply due to increasing the capacity for lipoprotein maturation or OM trafficking through transcriptional upregulation of these pathways. Even in *E. coli*, where we understand in molecular detail how the Cpx response is triggered by NlpE in response to OM lipoprotein biogenesis defects, there is still an incomplete understanding of how Cpx exerts its protective effects (27–29). One key role of Cpx appears to be protecting IM integrity to preserve the energy generating capacity of the cell (31, 54). Mislocalized OM-targeted lipoproteins can present a threat to IM integrity, making Cpx’s role in protecting the IM and in mitigating OM lipoprotein biogenesis defects congruent functions. Perhaps the BfmRS response similarly performs a primarily IM protective role in *A. baumannii*. Further work will be required to test this hypothesis.

In this study, we predicted all lipoproteins likely produced by *A. baumannii* ATCC 17978. Defects that we induced in OM lipoprotein biogenesis (*lgti* and *lnti*) resulted in consistent differential regulation of 44 lipoprotein genes. A block in trafficking OM-targeted lipoproteins causes them to mislocalized and accumulate in the IM. It is tempting to speculate that the downregulated genes encode for lipoproteins that are particularly toxic if mislocalized in the IM of *A. baumannii*. Little is currently known about the functions of the lipoproteins that we found were differentially regulated and more work will be needed to understand how their regulation relates to *A. baumannii* cell envelope homeostasis.

Why aren’t ESRs widely conserved? The OM components of *E. coli* and *A. baumannii* are practically identical: β-barrel OMPs, lipid A species (either decorated with a polysaccharide or not), phospholipids, and lipoproteins. The pathways for transporting and assembling these components are also conserved between the two organisms. Yet, no ESRs have been conserved. Apparently, a bespoke solution to stress sensing and mitigation has formed in each organism. Indeed, NlpE—despite being conserved—does not appear to be involved in a protective response to OM lipoprotein trafficking stress in *A. baumannii*. Our work advances our understanding of OM homeostasis in the important human pathogen *A. baumannii* and, in the case of NlpE, it also underscores that caution that should be taken when attempting to directly transplant a molecular model from one relatively well-understood species to another, distantly related bacterium without direct investigation.

## MATERIALS AND METHODS

### Bacterial strains, plasmids, and growth conditions

Strains and plasmids used in this study are listed in Tables S3 and S4, respectively. Chromosomal null alleles were generated using pAT02 recombineering, and their Kan^R^ cassettes were cured using pAT03 as previously described by (62). Strains were grown in Lennox broth or agar at 37°C. Media were supplemented with kanamycin (Kan; 25 µg/mL), carbenicillin (Carb; 50 µg/mL), or anhydrotetracycline (aTc; 0.5–10 ng/mL for induction of dCas9).

### Generation of knockout mutants using recombineering

All mutants were generated by recombineering in *A. baumannii* 17978 aTc-dCas9, as previously described (62) using oligos in Table S5 In brief, FRT-flanked kanamycin cassette and 1 kb of upstream and downstream regions of gene of interest (*nlpE*, *amsSR*, *baeR*, and *bfmRS*) were amplified from pKD4 and gDNA, respectively. PCR products were Gibson assembled into pBBR1MCS. The complete cassette with flanking regions of homology to the chromosome was amplified by PCR and purified. A total of 5 µg of linear recombineering product was introduced to 50 µL of electrocompetent *A. baumannii* expressing the recombinase from pAT02. Cells were recovered in LB for 90 min at 37°C, and recombinase expression was induced during recovery. Transformants were plated onto LB agar plates supplemented with kanamycin and incubated overnight at 37°C. Colonies were screened by PCR using primer pairs in the oligo list in Table S5. Colonies were screened for the loss of pAT02 by patching onto LB agar plates supplemented with carbenicillin. Unmarked mutants were generated by introducing pAT03 into Kan^R^ mutant strains and inducing expression of Flp recombinase to excise the kanamycin cassette, as previously described (62). Briefly, transformants were streaked onto LB agar plates containing IPTG and then subsequently streaked onto LB agar plates and patched onto LB plates containing kanamycin to check for excision of the cassette and carbenicillin to screen for loss of pAT03.

### Generation of *bfmRS* complement mutant using recombineering

Complement was generated using recombineering in *A. baumannii* 17978 aTc-dCas9 to replace *hisG* with the *bfmRS* operon. Briefly, FRT-flanked kanamycin cassette, *bfmRS* operon, and 1 kb upstream and downstream regions of *hisG* were amplified from pKD4 or gDNA. Gibson assembly was used to assemble the cassette containing *bfmRS* operon with the kanamycin cassette downstream, flanked on either end with 1 kb homology to the upstream and downstream regions of *hisG*, into pBBR1MCS. Recombineering was performed as previously described above with 5 µg of PCR construct. Transformants were selected on LB agar supplemented with kanamycin. Colonies were screened for *hisG* gene replacement by patching onto M63-sodium succinate (25 mM final concentration) plates. These minimal media do not contain histidine, and therefore, *hisG*^-^ mutants will not grow. Insertion of *bfmRS* operon was determined *via* colony PCR using oligos from Table S5. Auxotrophic mutants were patched onto LB media containing carbenicillin to screen for loss of pAT02.

### Construction gRNA plasmids for CRISPRi

Plasmid pYDE007 encoding a non-targeting sgRNA designed for *mrfp* as previously described (33) was PCR site-directed mutagenized to change the sgRNA to target *A. baumannii* 17978 *lgt*, *lnt*, *lolA,* and *lptD*. Guide RNA design parameters consisted of a targeting sequence 24 bp long 5′ to PAM site (NGG) close to the transcription start site of the target gene. The sgRNA was designed to target the non-template strand and has a 12-nt seed region unique to gene locus. The sgRNA sequence on pYDE007 was replaced *via* site-directed mutagenesis with oligonucleotides listed in Table S5.

### Efficiency of plating assays

The efficiency of plating (EOP) assays were used to determine the sensitivity of strains grown in the presence of aTc (in strains with aTc-dependent dCas9 expression). Assays were performed by preparing 10-fold serial dilutions of overnight cultures in 96-well microtiter plates before replica plating on LB agar plates with and without aTc inducer (concentration as noted) and then incubating plates overnight at 37°C.

### CRISPRi of target genes

Strains harboring non-targeting, *lgt*, or *lnt* gRNA-expressing plasmid were grown overnight in LB supplemented with carbenicillin. Strains were subcultured 1:100 in fresh LB broth with 25 ng/mL aTc and carbenicillin in 24-well plates. Cultures were grown at 37°C with continuous shaking until an A_600_ of ~0.4 was reached.

### RT-qPCR analysis

RNA was prepared from CRISPRi deplete cells as described by New England Biolabs Monarch Total RNA miniprep kit. qRT-PCR was performed with oligonucleotides listed in Table S5. Relative levels of RNA were calculated using the ΔΔCT method relative to the wild-type strain under non-targeting sgRNA conditions. Data are the averages from three independent biological replicates +/-the standard errors of the means (SEMs).

### RNA-seq analysis

RNA was prepared from CRISPRi deplete cells as described above. Illumina RNA sequencing with rRNA depletion was performed on each sample by SeqCenter in Pittsburgh, Pennsylvania. Data are from two biological replicates per strain. Analysis to calculate Log_2_ fold change (FC) and *P*-values were performed using CLC Workbench, and all data can be found in the supplementary. Raw Illumina reads from RNA-Seq are deposited in the NCBI Sequence Read Archive database under BioProject number PRJNA1148128.

### AlphaFold2 predictions

To obtain mature sequences of *A. baumannii* NlpE, the site of signal peptidase II cleavage was predicted with SignalP-6.0 (63). Mature NlpE was inputted into AlphaFold Google Colab notebook, which uses a simplified version of AlphaFold v2.3.2 (64). The program exported the model with the highest confidence (pTM).

### SignalP 6.0 predictions

To predict likely lipoproteins encoded by *A. baumannii* strain ATCC 17978, we used SignalP 6.0 (63) algorithm to assess each translated protein product. We first benchmarked SignalP 6.0 against proteins produced by *E. coli* strain MG1655 to predict all signal peptidase II processes proteins. A SignalP “LIPO(Sec/SPII)” score of >0.8 accurately predicted all experimentally validated lipoproteins and was used as the cut-off score for identifying likely *A. baumannii* lipoproteins (Sec/SPII or Tat/SPII).

## SUPPLEMENTAL MATERIAL

Supplemental figures and tables jb.00332-24-s0001.docx Fig. S1 and S2; Tables S3 to S5.

Table S1 jb.00332-24-s0002.xlsx lgti and lnti gene expression data.

Table S2 jb.00332-24-s0003.xlsx Predicted lipoproteins and their gene expression data during lgti and lnti.

ASM does not own the copyrights to Supplemental Material that may be linked to, or accessed through, an article. The authors have granted ASM a non-exclusive, world-wide license to publish the Supplemental Material files. Please contact the corresponding author directly for reuse.
