# Supplementary material for: The BfmRS stress response protects Acinetobacter baumannii against defects in outer membrane lipoprotein biogenesis
Source: J Bacteriol. 2024 Dec 11;207(1):e00332-24. doi: 10.1128/jb.00332-24 (PMC11784087; doi:10.1128/jb.00332-24)
Supplement: Supplemental figures and tables — Fig. S1 and S2; Tables S3 to S5. [file jb.00332-24-s0001.docx]

**Figure S1: BfmRS does not regulate *ompW* or *tolC*.** RNA was isolated from wild-type, *bfmRS,* and *bfmRS* complemented strains from three biological replicates at midlog phase. Expression of *tolC* (A1S_0535) and *ompW* (A1S_0292), was measured via RT-qPCR. Log_2_FC was calculated via the ΔΔCT method and expression was compared to the WT strain. A 2-way ANOVA was performed to compare normalized expression.

**Figure S2: *bfmR* expression is unchanged during OM lipoprotein biogenesis stress (*lgt*i).** RNA was isolated from *lgt* depleted cells (*lgti*) or non-targeting (NT) control from three biological replicates. Expression of *bfmR* (A1S_0748) and was measured via RT-qPCR. Log_2_FC was calculated via the ΔΔCT method and expression was compared to the WT strain under non-targeting conditions. A 2-way ANOVA was performed to compare normalized expression.

**Table S3: Strains used in this study**

| **Name** | **Genotype** | **Source** |
| --- | --- | --- |
| JM540 | *A. baumannii* 17978 *att*Tn7::*tetR*-*tetP*-*dcas9*-*rrnBT1*-T7 | (1) |
| JM572 | JM540 [psgRNA non-targeting control] | This study |
| JM574 | JM540 [psgRNA *lgt*-targeting] | This study |
| JM575 | JM540 [psgRNA *lnt*-targeting] | This study |
| JM663 | JM540 [psgRNA *lolA*-targeting] | This study |
| JM699 | JM540 [psgRNA *lptD*-targeting] | This study |
| JM675 | JM540 *nlpE::kan* | This study |
| JM679 | JM675 [psgRNA non-targeting control] | This study |
| JM685 | JM675 [psgRNA *lgt*-targeting] | This study |
| JM686 | JM675 [psgRNA *lnt*-targeting] | This study |
| JM680 | JM675 [psgRNA *lolA*-targeting] | This study |
| JM666 | JM540 Δ*amsSR* | This study |
| JM687 | JM666 [psgRNA non-targeting control] | This study |
| JM688 | JM666 [psgRNA *lgt*-targeting] | This study |
| JM689 | JM666 [psgRNA *lnt*-targeting] | This study |
| JM690 | JM666 [psgRNA *lolA*-targeting] | This study |
| JM624 | JM540 Δ*baeR* | This study |
| JM635 | JM624 [psgRNA non-targeting control] | This study |
| JM636 | JM624 [psgRNA *lgt*-targeting] | This study |
| JM684 | JM624 [psgRNA *lnt*-targeting] | This study |
| JM647 | JM624 [psgRNA *lolA*-targeting] | This study |
| JM704 | JM624 [psgRNA *lptD*-targeting] | This study |
| JM664 | JM540 Δ*bfmRS* | This study |
| JM681 | JM664 [psgRNA non-targeting control] | This study |
| JM683 | JM664 [psgRNA *lnt*-targeting] | This study |
| JM682 | JM664 [psgRNA *lolA*-targeting] | This study |
| JM701 | JM664 [psgRNA *lptD*-targeting] | This study |
| JM708 | JM540 *bfmRS-kan::hisG* | This study |
| JM713 | JM708 [pgsRNA non-targeting control] | This study |
| JM714 | JM708 [pgsRNA *lnt*-targeting] | This study |
| JM715 | JM708 [pgsRNA *lolA*-targeting] | This study |
| JM711 | JM664 *bfmRS-kan::hisG* | This study |
| JM716 | JM711 [pgsRNA non-targeting control] | This study |
| JM717 | JM711 [pgsRNA *lnt*-targeting] | This study |
| JM718 | JM711 [pgsRNA *lolA*-targeting] | This study |

**Table S4: Plasmids used in this study**

| **Plasmid** | **Source** |
| --- | --- |
| pYDE007 sgRNA non-targeting control (*mrfp*) | (1) |
| pYDE007 sgRNA *lgt*-targeting control | This study |
| pYDE007 sgRNA *lnt*-targeting control | This study |
| pYDE007 sgRNA *lolA*-targeting control | This study |
| pYDE007 sgRNA *lptD*-targeting control | This study |
| pBBR1MCS | (2) |
| pBBR1MCS *nlpE* kanamycin cassette | This study |
| pBBR1MCS *amsSR* kanamycin cassette | This study |
| pBBR1MCS *baeR* kanamycin cassette | This study |
| pBBR1MCS *bfmRS* kanamycin cassette | This study |
| pBBR1MCS *bfmRS*-kan *hisG* cassette | This study |
| pAT02 | (3) |
| pAT03 | (3) |
| pKD4 | (4) |

**Table S5: Oligonucleotides used in this study**

| **Name** | **Sequence (5’-3’)** | **Note** |
| --- | --- | --- |
| **Assembling sgRNA plasmids** | | |
| gRNA_SDM_R | ACTAGTATTATACCTAGGACTG | amplify pYDE007 to insert gRNA target sequence to replace the nontargeting *mrfp* sequence |
| Lgt_F | AAGAGGTCCTAGATGTATTGCGACGTTTTAGAGCTAGAAATAGC | amplify pYDE007 to insert gRNA target sequence specific to *lgt* to replace the nontargeting *mrfp* sequence |
| Lnt_F | AAGGTGCCAGTGCAAAACTGAACAGTTTTAGAGCTAGAAATAGC | amplify pYDE007 to insert gRNA target sequence specific to *lnt* to replace the nontargeting *mrfp* sequence |
| LolA_F | TACTCATTACAGGAGCAAGCACTGGTTTTAGAGCTAGAAATAGC | amplify pYDE007 to insert gRNA target sequence specific to *lolA* to replace the nontargeting *mrfp* sequence |
| LptD_F | CTGACTCAGCATAACTTGATTGTAGTTTTAGAGCTAGAAATAGC | amplify pYDE007 to insert gRNA target sequence specific to *lptD* to replace the nontargeting *mrfp* sequence |
| **Assembling kanamycin cassettes for recombineering** | | |
| pBBR1MCS_gibson_R | CCCAACTTAATCGCCTTGCAG | amplify pBBR1MCS plasmid backbone |
| pBBR1MCS_gibson_F | AGCTGTTTCCTGTGTGAAATTG | amplify pBBR1MCS plasmid backbone |
| pKD4kan_F | GATTGTGTAGGCTGGAGCTG | amplify kanamycin cassette with FRT sites |
| pKD4kan_R | CATATGAATATCCTCCTTAGTTCC | amplify kanamycin cassette with FRT sites |
| BaeR_pBBR1MCS_F | TGTGCTGCAAGGCGATTAAGTTGGGGTCAATAGTCAGGTCGTAGG | amplify 1kb upstream *baeR* with pBBR1MCS adapter |
| BaeR_pKD4kan_R | CGAAGCAGCTCCAGCCTACACAATCGGGCCATTTTTCCTTTTATTTAAC | amplify 1kb upstream *baeR* with kanamycin cassette adapter |
| BaeR_pKD4kan_F | AGGAACTAAGGAGGATATTCATATGTAATTGCTTAGAAAAGTTATGCTG | amplify 1kb downstream *baeR* with kanamycin cassette adapter |
| BaeR_pBBR1MCS_R | TAACAATTTCACACAGGAAACAGCTTGAATGTCATATCCGTTTAGG | amplify 1kb downstream *baeR* with pBBR1MCS adapter |
| BaeR_kan_F | GTCAATAGTCAGGTCGTAGG | amplify entire kanamycin cassette with *baeR* flanking homology for recombineering |
| BaeR_kan_R | TGAATGTCATATCCGTTTAGG | amplify entire kanamycin cassette with *baeR* flanking homology for recombineering |
| BfmR_pBBR1MCS_F | TGTGCTGCAAGGCGATTAAGTTGGGTTGAACATCAATACCTTCTAAGCC | amplify 1kb upstream *bfmR* with pBBR1MCS adapter |
| BfmR_pKD4kan_R | CGAAGCAGCTCCAGCCTACACAATCATCATTGCCCCTATAAATCTCATTAC | amplify 1kb upstream *bfmR* with kanamycin cassette adapter |
| BfmS_pKD4kan_F | AGGAACTAAGGAGGATATTCATATGGGTGCTTTTTTTATTGCTTCATTTATAAC | amplify 1kb downstream *bfmS* with kanamycin cassette adapter |
| BfmS_pBBR1MCS_R | TAACAATTTCACACAGGAAACAGCTCCATTCTGAATTAATTCAGAAGTTACAAC | amplify 1kb downstream *bfmS* with pBBR1MCS adapter |
| BfmR_kan_F | TTGAACATCAATACCTTCTAAGCC | amplify entire kanamycin cassette with *bfmR* flanking homology for recombineering |
| BfmS_kan_R | CCATTCTGAATTAATTCAGAAGTTACAAC | amplify entire kanamycin cassette with *bfmS* flanking homology for recombineering |
| hisG_up_F | GCTGCAAGGCGATTAAGTTGGGGCTTGTCAAAACAATGCG | amplify 1kb upstream *hisG* with pBBR1MCS adapter |
| hisG_up_R | TTAACTTACTCGCTTAATTTTGGC | amplify 1kb upstream *hisG* |
| hisG_down_F | AACTAAGGAGGATATTCATATGTAAAAGTAATTTCCTATCATGC | amplify 1kb downstream *hisG* with kanamycin cassette adapter |
| hisG_down_R | CAATTTCACACAGGAAACAGCTCAATCTCAGCTTTAGGCAAT | amplify 1kb downstream *hisG* with pBBR1MCS adapter |
| BfmRS_F | CAAAATTAAGCGAGTAAGTTAATTGTCGGGAGATAGCATACCAAAG | amplify *bfmRS* operon including promoter with *hisG* adapter |
| BfmRS_R | AGCAGCTCCAGCCTACACAATCGCAAACCTATTTTGGAACCTGATG | amplify *bfmRS* operon including promoter with kanamycin cassette adapter |
| hisG_F | GCTTGTCAAAACAATGCG | Amplify entire *bfmRS-kan* complement construct with *hisG* flanking homology for recombineering |
| hisG_R | CAATCTCAGCTTTAGGCAAT | Amplify entire *bfmRS-kan* complement construct with *hisG* flanking homology for recombineering |
| amsSR_pBBR1MCS_F | TGTGCTGCAAGGCGATTAAGTTGGGCTAAAATCACCCAGCCCGG | amplify 1kb upstream *amsS* with pBBR1MCS adapter |
| amsSR_pKD4kan_R | CGAAGCAGCTCCAGCCTACACAATCGTTTTGGATAGGGCTGC | amplify 1kb upstream *amsS* with kanamycin cassette adapter |
| amsSR_pKD4kan_F | AGGAACTAAGGAGGATATTCATATGTAATTTAAGTGAAGATTGTATTTAAAAAGC | amplify 1kb downstream *amsR* with kanamycin cassette adapter |
| amsSR_pBBR1MCS_R | TAACAATTTCACACAGGAAACAGCTTAAAAAGGTCAGTCCGATC | amplify 1kb downstream *amsR* with pBBR1MCS adapter |
| amsSR_kan_F | CTAAAATCACCCAGCCCGG | amplify entire kanamycin cassette with *amsS* flanking homology for recombineering |
| amsSR_kan_R | TAAAAAGGTCAGTCCGATC | amplify entire kanamycin cassette with *amsR* flanking homology for recombineering |
| NlpE_pBBR1MCS_F | TGTGCTGCAAGGCGATTAAGTTGGGCTCCCGGTGTGGTAATTA | amplify 1kb upstream *nlpE* with pBBR1MCS adapter |
| NlpE_pKD4kan_R | CGAAGCAGCTCCAGCCTACACAATCAATGAATAAAACCTCAAGAAAATAAATATAAAG | amplify 1kb upstream *nlpE* with kanamycin cassette adapter |
| NlpE_pKD4kan_F | AGGAACTAAGGAGGATATTCATATGTAATCTATTTTTCAATATAAATAAAAAACCCGC | amplify 1kb downstream *nlpE* with kanamycin cassette adapter |
| NlpE_pBBR1MCS_R | TAACAATTTCACACAGGAAACAGCTGTCACCAAGCTGGTAAAGTAA | amplify 1kb downstream *nlpE* with pBBR1MCS adapter |
| NlpE_kan_F | GCTCCCGGTGTGGTAATTA | amplify entire kanamycin cassette with *nlpE* flanking homology for recombineering |
| NlpE_kan_R | GTCACCAAGCTGGTAAAGTAA | amplify entire kanamycin cassette with *nlpE* flanking homology for recombineering |
| **qPCR** | | |
| clpX_F | GCGTTTGAAAGTCGGGCAAT | amplify *clpX* (5) |
| clpX_R | CCATTGCAAACGGCACATCT | amplify *clpX* (5) |
| A1S2729_F | TACAGCGTTTGACTGCCGAT | amplify *lolA* |
| A1S2729_R | TTCCCCGGACGTTCAACTTT | amplify *lolA* |
| A1S0292_F | GTGGTAAAGGCGGACCATGA | amplify *ompW* |
| A1S0292_R | ACGCGCTACTTTCTGACCAT | amplify *ompW* |
| A1S0535_F | AAGAGCCACAGCGTTTACCA | amplify *tolC* |
| A1S0535_R | CTTTTCTCAAGCGAAGCTCGG | amplify *tolC* |
| A1S0460_F | CCATGTGTACCGACAGGCTT | amplify *lgt* |
| A1S0460_R | GATCTGTAACCGCACGACCA | amplify *lgt* |
| A1S0373_F | GGTTATGCGCCGCTATTTGG | amplify *lnt* |
| A1S0373_R | AACAGGGCAGCAGGGATAAC | amplify *lnt* |
| A1S1546_F | TGAACCAAGAAACTGGGCGT | amplify *lptD* |
| A1S1546_R | GAGGATACCTGTAGTGCGGC | amplify *lptD* |

**SUPPLEMENTAL REFERENCES**

1. Bai J, Dai Y, Farinha A, Tang AY, Syal S, Vargas-Cuebas G, van Opijnen T, Isberg RR, Geisinger E. 2021. Essential Gene Analysis in *Acinetobacter baumannii* by High-Density Transposon Mutagenesis and CRISPR Interference. J Bacteriol 203:e0056520.

2. Kovach ME, Phillips RW, Elzer PH, Roop RM, 2nd, Peterson KM. 1994. pBBR1MCS: a broad-host-range cloning vector. Biotechniques 16:800-2.

3. Tucker AT, Nowicki EM, Boll JM, Knauf GA, Burdis NC, Trent MS, Davies BW. 2014. Defining gene-phenotype relationships in *Acinetobacter baumannii* through one-step chromosomal gene inactivation. mBio 5:e01313-14.

4. Datsenko KA, Wanner BL. 2000. One-step inactivation of chromosomal genes in *Escherichia coli* K-12 using PCR products. Proc Natl Acad Sci U S A 97:6640-5.

5. Anderson SE, Chin CY, Weiss DS, Rather PN. 2020. Copy Number of an Integron-Encoded Antibiotic Resistance Locus Regulates a Virulence and Opacity Switch in *Acinetobacter baumannii* AB5075. mBio 11:e02338-20.
